# Flicker Regularity Is Crucial for Entrainment of Alpha Oscillations

**DOI:** 10.3389/fnhum.2016.00503

**Published:** 2016-10-13

**Authors:** Annika Notbohm, Christoph S. Herrmann

**Affiliations:** ^1^Experimental Psychology Lab, Center for Excellence ‘Hearing4all’, European Medical School, Carl von Ossietzky UniversityOldenburg, Germany; ^2^Research Center Neurosensory Science, Carl von Ossietzky UniversityOldenburg, Germany

**Keywords:** EEG, entrainment, alpha, flicker, SSVEP, oscillations, visual perception

## Abstract

Previous studies have shown that alpha oscillations (8–13 Hz) in human electroencephalogram (EEG) modulate perception via phase-dependent inhibition. If entrained to an external driving force, inhibition maxima and minima of the oscillation appear more distinct in time and make potential phase-dependent perception predictable. There is an ongoing debate about whether visual stimulation is suitable to entrain alpha oscillations. On the one hand, it has been argued that a series of light flashes results in transient event-related responses (ERPs) superimposed on the ongoing EEG. On the other hand, it has been demonstrated that alpha oscillations become entrained to a series of light flashes if they are presented at a certain temporal regularity. This raises the question under which circumstances a sequence of light flashes causes entrainment, i.e., whether an arrhythmic stream of light flashes would also result in entrainment. Here, we measured detection rates in response to visual targets at two opposing stimulation phases during rhythmic and arrhythmic light stimulation. We introduce a new measure called “behavioral modulation depth” to determine differences in perception. This measure is capable of correcting for inevitable artifacts that occur in visual detection tasks during visual stimulation. The physical concept of entrainment predicts that increased stimulation intensity should produce stronger entrainment. Thus, two experiments with medium (Experiment 1) and high (Experiment 2) stimulation intensity were performed. Data from the first experiment show that the behavioral modulation depth (alpha phase-dependent differences in detection threshold) increases with increasing entrainment of alpha oscillations. Furthermore, individual alpha phase delays of entrained alpha oscillations determine the behavioral modulation depth: the largest behavioral modulation depth can be found if targets presented during the minimum of the entrained oscillation are compared to those presented during the maximum. In the second experiment stimulation with higher light intensity during both rhythmic and arrhythmic stimulation lead to an increased behavioral modulation depth, supposedly as a consequence of stronger entrainment during rhythmic stimulation. Altogether, our results reveal evidence for rhythmic and arrhythmic visual stimulation to induce fundamentally different processes in the brain: we suggest that rhythmic but not arrhythmic stimulation interacts with ongoing alpha oscillations via entrainment.

## Introduction

**Figure 1:**
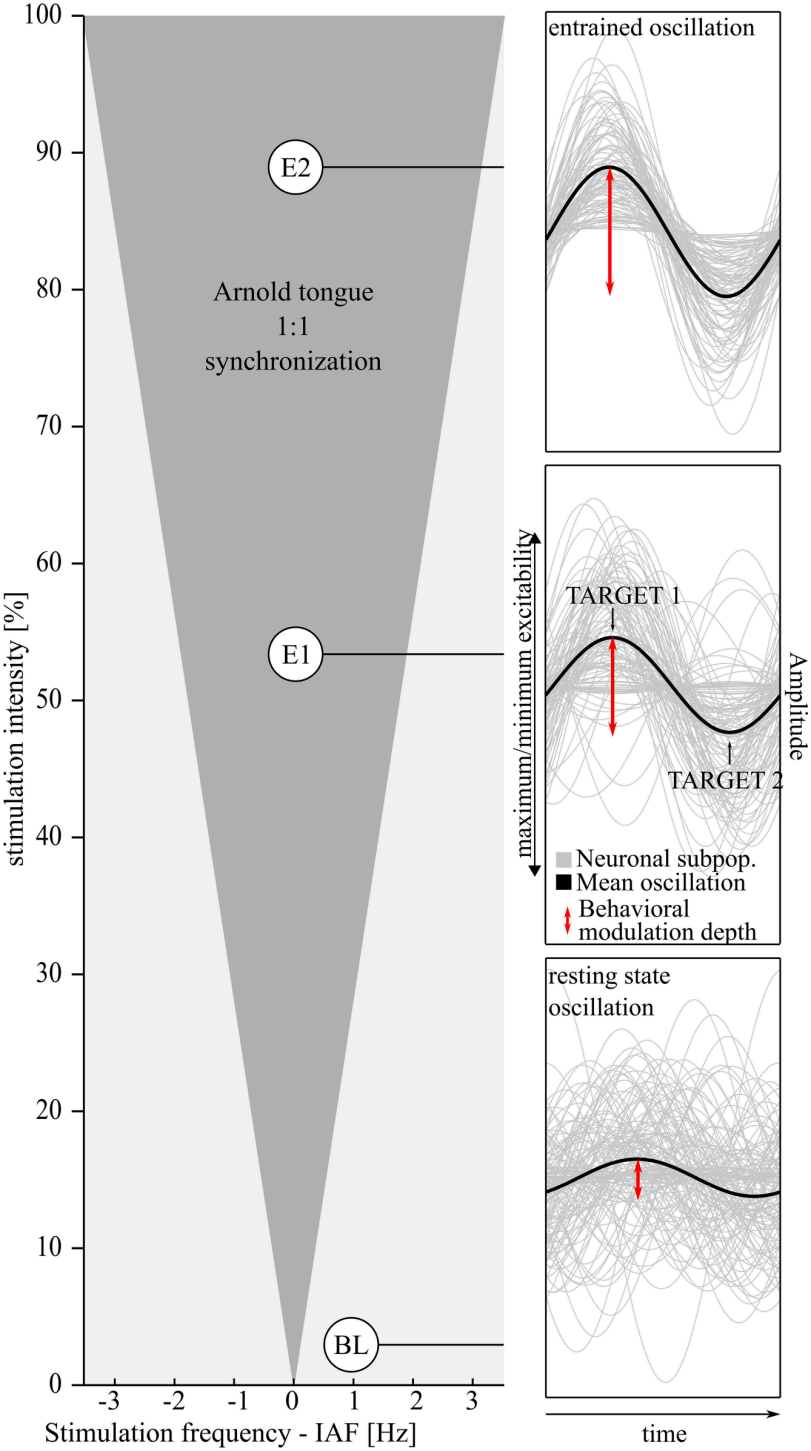
**The hypothesized effect of visual entrainment on alpha oscillations and, as a consequence, on the behavioral modulation depth**. **Left**: The Arnold tongue (Pikovsky et al., 2003) predicts the degree of entrainment as a function of frequency and intensity of the flickering light. Close to the individual alpha frequency (IAF) and with increasing light intensity entrainment (gray shaded triangular area) becomes more likely. Two different stimulation intensities (Experiments 1 and 2, E1 and E2, reported here) are expected to result in different degrees of entrainment compared to baseline (BL) see also Notbohm et al. (2016). The **right** panels depict an increasing degree of entrainment of alpha subpopulations from bottom to top with the mean oscillatory phase in black. The behavioral modulation depth (red arrows) is expected to increase accordingly. **Bottom**: Outside the suggested Arnold tongue, alpha oscillations not entrained—just as in the resting state. Medium stimulation intensity (Experiment 1): due to entrainment, phases are aligned to the external stimulation. Amplitude extrema (peak/trough) reflect phases of maximum/minimum excitability and become more distinct in time during entrainment. Top panel: this effect is expected to be increased in Experiment 2 (E2, higher stimulation intensity).

A near-threshold visual light flash may in some occasions be perceived by an observer, in others not, although the physical stimulus is identical in both instances. Several studies have revealed a decisive role of oscillatory neural activity in this context. More precisely, the oscillatory phase (Klimesch et al., 2007; Mathewson et al., 2009; Haegens et al., 2011b) and amplitude (Ergenoglu et al., 2004; Hanslmayr et al., 2005) in the alpha range (8–13 Hz) have been shown to correlate with detectability of the visual stimulus. A higher amplitude of the alpha frequency band measured in electroencephalogram (EEG) is based on stronger phase synchrony between neural populations (Hanslmayr et al., 2007a; Klimesch et al., 2007). Thus, EEG-phase angle dependencies come more into play when the optimal phase for perception coincides in time for a large amount of neurons (Figure 1, right panels).

Via external visual stimulation, it has been suggested that neuronal populations that are involved in generating intrinsic alpha oscillations can be forced to oscillate at the externally applied frequency, but this mechanism is still a matter of debate. In a previous experiment, we found that rhythmic visual stimulation can synchronize ongoing alpha oscillations (Notbohm et al., 2016). The phase of the alpha oscillation was locked to the external visual oscillator if the stimulation frequency was close to the intrinsic individual alpha frequency (Tass et al., 1998; Halbleib et al., 2012). Additionally, the light intensity of the external driving oscillator correlated positively with the degree of phase coupling between internal and external phase. Plotted as a function of stimulation frequency and phase, phase locking was strongest close to the individual alpha frequency and increased with increasing light intensity. The resulting pattern resembled the physical concept of entrainment, called the Arnold tongue (Tass et al., 1998; Pikovsky et al., 2003). This pattern was not found if the light stimulation was arrhythmic (Notbohm et al., 2016). These findings reveal evidence that entrainment is the underlying mechanism of the steady-state visual evoked potential (SSVEP). The SSVEP defines the brain response in the occipito-parietal cortex that appears during rhythmic light stimulation (Hillyard et al., 1997; Herrmann, 2001; Roberts and Robinson, 2012). SSVEPs seemed to differ from brain responses during arrhythmic stimulation, where the brain responses were rather independent from the ongoing brain oscillations (Notbohm et al., 2016). This theory is supported by Parkes et al. (2004), who found that rhythmic but not arrhythmic stimulation resulted in decreased blood-oxygen level dependent (BOLD) responses. BOLD responses, measured during functional MRI (magnetic resonance imaging), are known to correlate negatively with the amplitude of alpha oscillations (Hillyard et al., 1997; Parkes et al., 2004). Thus, rhythmic but not arrhythmic stimulation led to an actual interaction with the ongoing oscillation.

Capilla et al. (2011), on the contrary, showed that SSVEPs resembled a synthetic signal where several isolated event-related responses (ERPs) were superimposed with inter-trial intervals as determined by the stimulation frequency. They concluded that rhythmic and arrhythmic stimulation resulted in the same responses in a way that—independently of stimulus regularity—a single light flash always results in an ERP. A sequence of ERPs resembles the alpha rhythm if induced at a similar frequency. As opposed to entrainment, however, ERP generation results in enhanced EEG power, while entrainment rather shifts ongoing oscillations toward the external frequency of the driving force (Parkes et al., 2004). Entrainment could happen without power enhancement if only the phase of the intrinsic oscillations is aligned to the external driving force. If, however, multiple oscillators are recorded in EEG, their aligned phases add up and appear like an enhanced amplitude. Moreover, entrainment can only occur if a driving force couples to an oscillator, that generates self-sustained oscillations, whereas ERPs can be generated also by randomly firing cells (Pikovsky et al., 2003).

Considering the findings on alpha oscillations, perception and entrainment as a whole, we here hypothesize that if perception is modulated by the alpha phase and if alpha phase can be modulated via entrainment, then entrainment—but not superposition—are expected to lead to changes in perception.

The relation between entrainment and perception has been addressed in previous studies. A series of visual rhythmic stimuli resulted in modulated detection rates of near threshold stimuli, depending on the phase angle of the stimulation they were presented at. Mathewson et al. (2011) showed that targets presented after a train of rhythmic visual stimuli were more likely to show phase dependent-detection rates after an extended stimulation train, whereas a short stream (four stimuli) resulted in a weaker phase-dependency for target detection. The experiment generally corroborates the theory of entrainment, because phase-locking between stimulation phase and EEG phase resulted in predictable phase-dependent detection rates.

Interestingly, a subsequent experiment showed that the observed effect of phase-dependent target detection can also be gained via arrhythmic stimulation (Mathewson et al., 2012), agreeing with Capilla et al. (2011) that both types of stimulation result in the same kind of brain response. Mathewson et al. (2012), however, suggest temporal attention to be the underlying mechanism of entrainment, whereas Capilla et al. (2011) suggest superimposed ERPs to explain both types of signals. Temporal attention occurs if a temporal concept is provided that allows prediction, as it is the case when presenting stimuli at a certain rhythm (Nobre et al., 2007).

This leads to the question: is rhythmicity crucial for entrainment? To investigate whether arrhythmic stimulation—just as rhythmic stimulation—can produce entrainment, we designed a behavioral paradigm that allows a distinction between the two. We introduce a new measure of behavioral modulation depth that combines both types of stimulation in a single measure. In order to relate to the physical concept of the Arnold tongue, the behavioral measure was chosen such that it is capable of controlling for several confounds: pupil size differences between conditions (Cheng et al., 2006), contrast differences and differing impact of temporal attention (Lasley and Cohn, 1981; Nobre et al., 2007; Rolke and Hofmann, 2007).

Targets were presented at two opposing phase angles during rhythmic or arrhythmic stimulation. If alpha oscillations are entrained during rhythmic stimulation, the detection threshold will be modified phase-dependently, such that the perception threshold for target detection differs from stimulation-phase dependent the perception threshold during the arrhythmic stimulation.

In order to validate the newly introduced measure of modulation depth, we investigated the relationship between modulation depth and EEG measures of entrainment. We expect EEG measures of the alpha oscillation, namely the inter-trial coherence and the alpha phase at target onset, to predict the behavioral modulation depth. With increasing phase-coupling and at optimal phases (see Figure 1, right two top panels), the modulation depth should increase (during entrainment).

Differences in the phase-dependent detection rates after rhythmic and arrhythmic stimulation, however, were also described by Mathewson et al. (2012) and might be due to temporal attention that is weaker but also present during arrhythmic stimulation (Nobre et al., 2007). Thus, after evaluation of the behavioral measure of the modulation depth, in a second experiment we stimulated subjects with higher luminance when performing exactly the same task. Based on the concept of the Arnold tongue, higher stimulation intensity is expected to result in stronger entrainment during the rhythmic conditions (compare Figure 1 top right and middle right panel), but not during the arrhythmic conditions. Subjects are thus expected to show a stronger modulation of the detection threshold (increased modulation depth, red arrows in Figure 1) as compared to Experiment 1 (lower intensity). If the same fundamental mechanism explained both—rhythmic and arrhythmic stimulation (either via temporal attention or superposition of ERPs)—no changes of the modulation depth between the two Experiments would be expected.

## Methods

### Subjects

In Experiment 1, 20 healthy students (25.4 ± 4.2 years, 10 females) from the University of Oldenburg participated. All subjects gave written informed consent before their participation. The experimental protocol was approved by the ethics committee of the University of Oldenburg and was conducted in accordance with the Declaration of Helsinki. Three subjects' data sets had to be discarded. For two of them the 50% threshold in the behavioral target detection task was outside of the examined range of light intensities (one above and one below). The third subject showed a sudden impedance drift during the EEG recording. In Experiment 2, 20 healthy students (23.2 ± 2.4 years, 12 females) from the University of Oldenburg participated; none of them participated in the first experiment. The datasets of six subjects had to be excluded, because the 50% threshold of the detection task was above the examined range. The number of excluded subjects exceeds that of Experiment 1 probably due to the higher luminance in Experiment 2. Increased luminance causes pupil downsizing (Spring and Stiles, 1948; Watson and Yellott, 2012), which in turn is accompanied by decreased light sensitivity (Troland, 1917).

Participants in the remaining samples had normal or corrected to normal vision and reported no psychiatric disorders, epilepsy in family history or febrile convolutions during childhood. According to the Edinburgh handedness inventory (Oldfield, 1971), all subjects were right-handed.

### Experiments 1 and 2

The procedure was exactly the same for both experiments. The only difference was the light intensity of the visual flicker (Experiment 1: 118 cd/m^2^, Experiment 2: 262 cd/m^2^). The target light intensity, frequency of the flicker, as well as any other parameter (see below) was identical in both experiments.

### Stimuli and procedure

**Figure 2:**
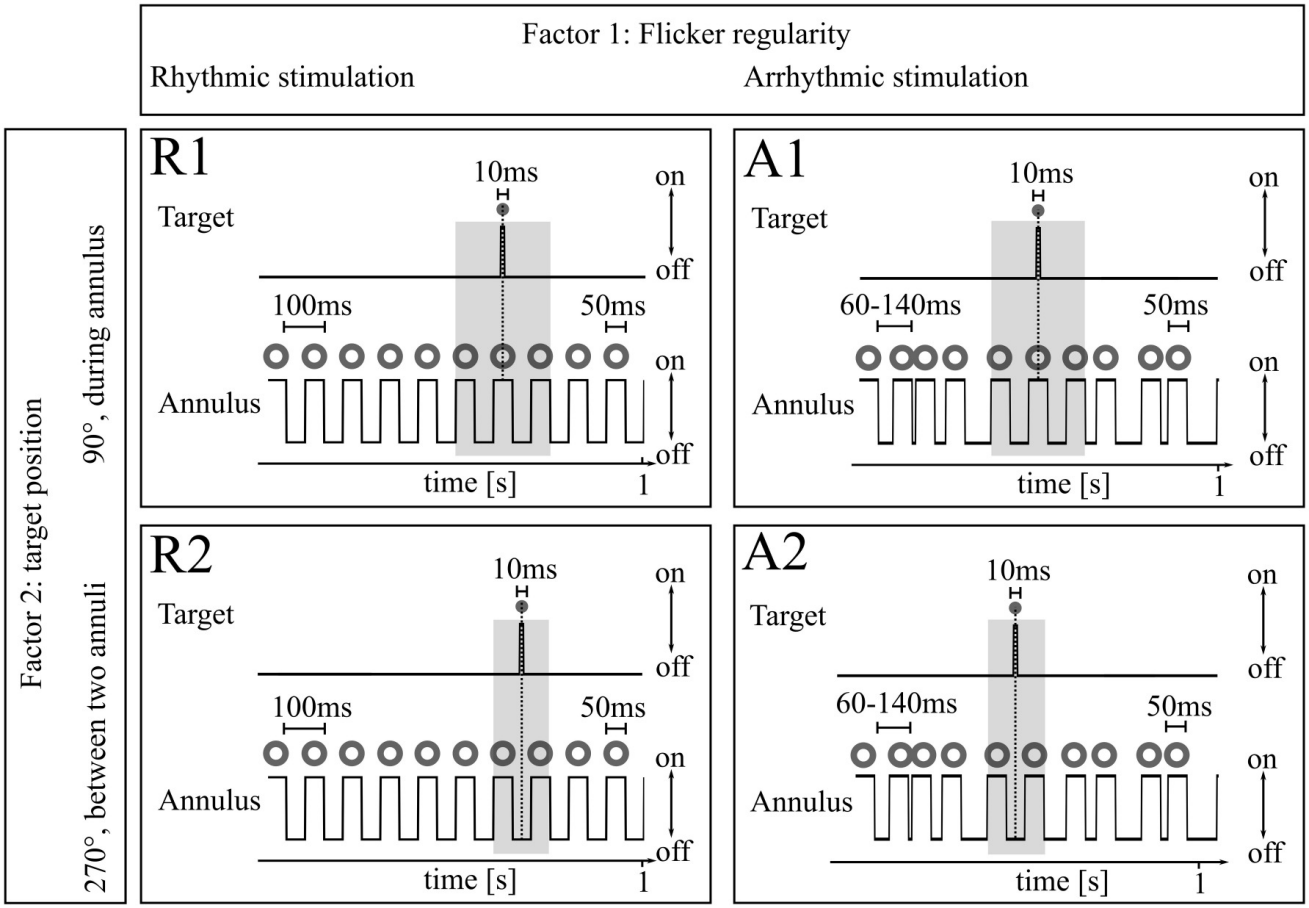
**Experimental design**. Both Experiments 1 and 2 consisted of the same experimental 2 × 2 paradigm with factors (1) Flicker regularity and (2) Target position. Factor 1: As flicker stimulus, an annulus flickered with 118 cd/m^2^ (262 cd/m^2^ in Experiment 2) either rhythmically (50 ms on, 50 ms off; 10 Hz) or arrhythmically (50 ms on, 10–90 ms off). Factor 2: Target position. Targets were presented during continuous flicker stimulation every 3–5 s in the center of the flickering annulus either during the annulus presentation (R1 and A1) or between two annuli (R2 and A2) for 10 ms in eight different light intensities. Subjects were asked to press a button whenever a target was detected. The gray boxes mark time periods around the target where inter-annulus intervals were kept constant between referring R/A conditions (50 ms; R1/A1 and R2/A2, respectively).

**Figure 3:**
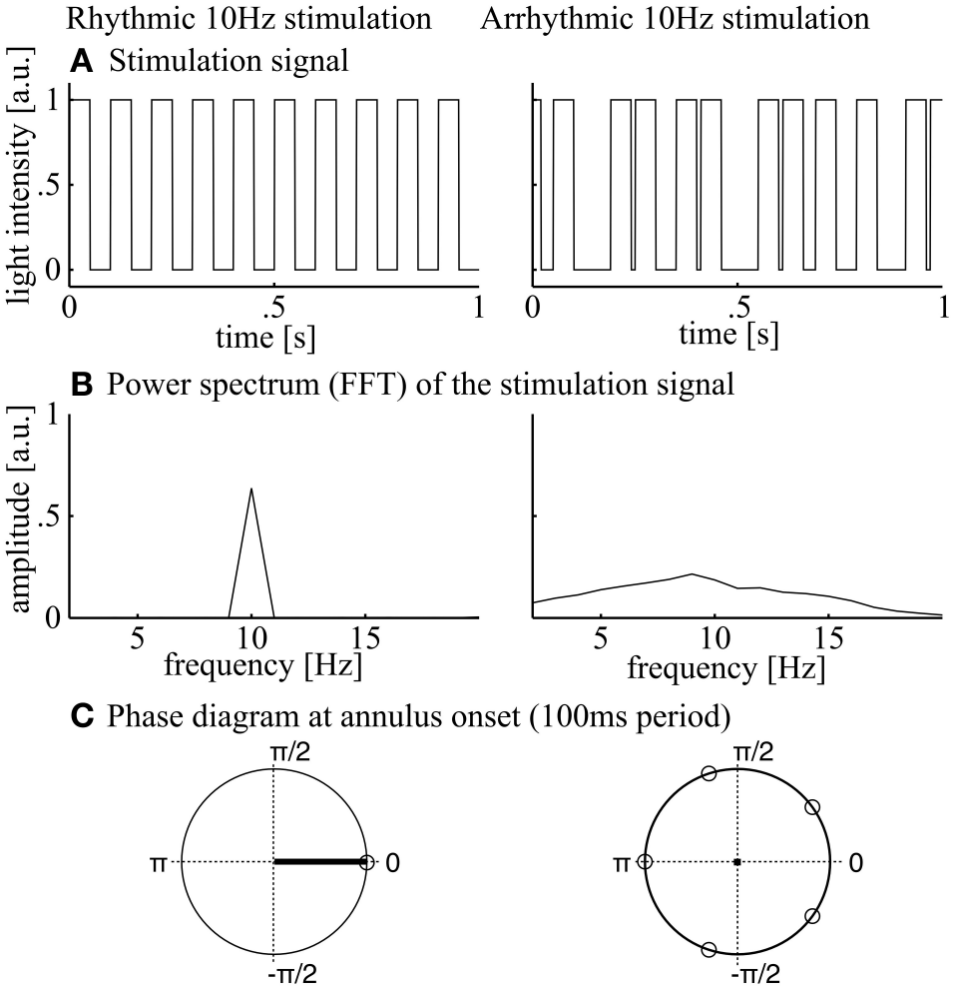
**Stimulation signals**. During rhythmic 10 Hz stimulation (left panels), square wave stimulation (annulus on or off, **A**) resulted in a clear peak at 10 Hz when regarding the power spectrum. The 10 Hz phase at annulus onset was 0 and the inter-trial phase coherence was 1 as indicated by the mean resultant length (black line in **C**, left panel). During arrhythmic stimulation (**A**, right panel), spectral power of the flicker was distributed over a range of frequencies around 10 Hz (**B**, right panel). **(C**) The phase angle at light onset was equally distributed over the unit cycle and the inter-trial phase coherence was 0 as indicated by the mean resultant length (black dot in **C**, right panel).

Subjects were seated in a dark sound-attenuated EEG chamber, 60 cm from a 24″ monitor (Samsung SyncMaster P2470) with a refresh rate of 100 Hz. The flicker was generated by a flashing annulus, located in the center of the screen at a visual angle of 2° (outer margin and 1° inner margin, see Figure 2) with a light intensity of 118 cd/m^2^ (262 cd/m^2^ in Experiment 2). To produce a 10 Hz rhythm in the rhythmic conditions, the annulus was presented for 50 ms followed by a 50 ms black screen (Figure 2). The experiments were structured in a 2 × 2 design with factors rhythmicity [rhythmic (R), arrhythmic (A)] and target position [90 (1)/270° (2), see Figure 2]. For the arrhythmic condition, the duration of the black screen was jittered between 10 and 90 ms (mean = 50 ms), while the flicker-on phases were kept at 50 ms to maintain constant luminous energy in both conditions (rhythmic/arrhythmic, see Figure 3 for stimulation details).

The same jitter duration never appeared twice in a row to preclude potential short-term entrainment, except for the target presentation period: before and after target presentation a black screen was always presented for 50 ms to keep light conditions constant around the target and preclude differences in target perception due to backward or forward masking (Eriksen, 1966) between the rhythmic and the arrhythmic target presentation. Targets were presented in the center of the flicker annulus at a visual angle of 1° (outer margin) for a duration of 10 ms either during presentation of an annulus or between two annuli (see Study design, Figure 2) and could appear at eight different light intensities between 0 and 262 cd/m^2^ in logarithmic steps. Each target presentation was repeated 20 times, resulting in 160 target presentations per condition, 640 targets in total. The experiment was written in MATLAB R2012b (The MathWorks Inc., Natick, MA, USA) using the Psychophysics Toolbox extensions (Brainard, 1997; Kleiner et al., 2007).

### Study design

The experiments were structured in a 2 × 2 block design with the factors flicker regularity (rhythmic/arrhythmic) and target position [peak (90°, 1)/trough (270°, 2) of stimulation phase] to modulate detection rates. Each of these four conditions was presented in a separate block with continuous flicker presentation and targets appearing every 4 ± 0.58 s (within the range of 3–5 s). The block order was randomized between subjects. No effect was found for the order [one-way ANOVA *F*_(1, 3)_ = 1.08, *p* = 0.36]. Each condition was repeated two times, resulting in 8 × 5 min blocks. After four blocks, the experiment was paused for at least 3 min, 1 min after each block. In the beginning and after the 3 min pause in the middle of the experiment, a short 1 min adaptation block was presented. Subjects were advised to press a button whenever a target (in the center of the flicker annulus) was detected. The ongoing flicker was unaffected by the button press. Before the actual experiment, 2 min resting state with eyes closed was recorded. Subsequently, a feedback block was presented for the subjects to learn the task. In contrast to the actual experiment, here subjects received feedback after each target, depending on whether the target was detected (green happy smiley), missed (1500 ms after target presentation; red sad smiley) or when subjects pressed a button when no target was shown during the preceding 1500 ms. The feedback loop was repeated until at least 80% of the test-targets (light intensities from 60 to 262 cd/m^2^) were detected.

### EEG recording

Electroencephalogram (EEG) was recorded during the whole experiment at 32 Ag–AgCl electrodes, mounted in an elastic cap (Easycap, Falk Minow, Munich, Germany) with an extended 10–20 system layout, referenced to the nose. FPz was used as ground electrode. As Pz has been described to capture the peak of alpha power in the parieto-occipital region, we chose this electrode for further analysis (Ergenoglu et al., 2004). Impedances were kept below 10 kΩ. EEG was recorded using a 16-bit Brain Amp DC amplifier and Brain Vision Recorder software (Brain Products GmbH, Gilching, Germany) with an online band pass filter of 0.016–250 Hz and a sampling rate of 1000 Hz. EEG was amplified in the range of ±16.384 μV at a resolution of 0.5 μV/bit. We recorded stimulus markers and responses were together with the EEG using Brain Vision recorder and stored data for further offline analysis.

Via photo diode we monitored the temporal accuracy of the flicker during stimulation. No inaccuracies were detected during measurements.

### Data analysis

#### Behavioral analysis

For the behavioral data, psychometric functions were fitted to the eight light intensities of the target and the detection rates. This was done for each of the four conditions and for each subject separately using a sigmoid function. This way, the 50% detection threshold was determined, resulting in four behavioral outcomes per subject. Due to confounding contrast differences between target positions 1 and 2 (R1 and R2; A1 and A2, respectively) and different levels of temporal expectation (Cravo et al., 2013) between rhythmic and arrhythmic stimulation (Figure S1B), the individual modulation depth was calculated via the following formula:

$$\begin{array}{l} Moldulation\;depth=abs\left(\frac{th\text{R}1}{th\text{R}2}-\frac{th\text{A}1}{th\text{A}2}\right) \end{array}$$

with *th* being the 50% detection threshold from the psychometric function for the conditions R1 and R2 (rhythmic stimulation, target positions 1 and 2) and A1 and A2 (arrhythmic stimulation, target positions 1 and 2, see also Figure 2). Please find further details for the derivation of this formula in the Supporting Information.

#### EEG analysis

EEG data were analyzed using MATLAB based EEGlab extensions (Delorme and Makeig, 2004). For preprocessing of the EEG data, we first visually inspected the data for eye blinks. If eye blinks affected the time period between −200 and 0 ms before target presentation, trials were ignored for further analysis. Then, a windowed sinc type I linear phase FIR filter (band pass: 1–20 Hz, filter order: 290) was applied to the data (Widmann and Schröger, 2012).

EEG amplitude spectra were calculated from resting state data and stimulation data using FFTs on 2 s sequences with an overlap of 1 s after band pass filtering between 1 and 20 Hz. We applied a hanning window and corrected for 1/f bias. For the induced spectrum (stimulated data), spectra were calculated for each single sequence and averaged subsequently. The evoked spectra were calculated by averaging sequences in the time domain and calculating an fft on averaged data subject wise.

To determine the inter-trial phase coherence (ITC), data were epoched from 200 ms before the onset to the onset of the flicker annulus that precedes a target presentation in condition R1 and R2 (rhythmic stimulation, target positions 90° and 270°, see Mathewson et al., 2009). The imaginary part of a Fast Fourier Transform (FFT) was then calculated at 10 Hz, no additional window function was applied. In the next step, the inter-trial coherence (ITC) was determined using the CircStat toolbox (Berens, 2009) by computing the absolute value of the mean phase angle for conditions R1 and R2.

To further determine the phase angle at target onset, the above described EEG data processing was repeated for the 200 ms interval right before target onset of condition 1 (R1). This period is most likely unaffected by target related movement artifacts and has previously been used to determine the phase at target onset (Mathewson et al., 2009). Instead of the ITC we now calculated the mean phase angle again using the CircStat toolbox.

#### Statistical analysis

A linear correlation was calculated for the ITC and the behavioral modulation depth per subject. Therefore, we took the minimum ITC from the two values gained from conditions R1 and R2. We here chose to take the minimum of the two, because if only during one of the two conditions entrainment was strong (high ITC), but in the other, for whatever reason, ITC was rather low, behavioral modulation depth could not be explained by phase depended behavior due to entrainment. The two values were anyhow highly correlated (*R*^2^ = 0.58, *p* < 0.001), which justifies taking the minimum of the two conditions.

Furthermore, a sinusoidal model was fitted to the behavioral modulation depth as a function of individual alpha phase angle at target onset (R1). In an iterative procedure, the parameters b_0_ and b_1_ (offset and amplitude) were estimated.

Henry and Obleser (2012) showed that subjects have an individual EEG phase delay with regard to the stimulus phase. Thus, targets were not necessarily presented at 90° and 270° of the EEG oscillation, just because this was the case for the stimulation phase.

**Figure 4:**
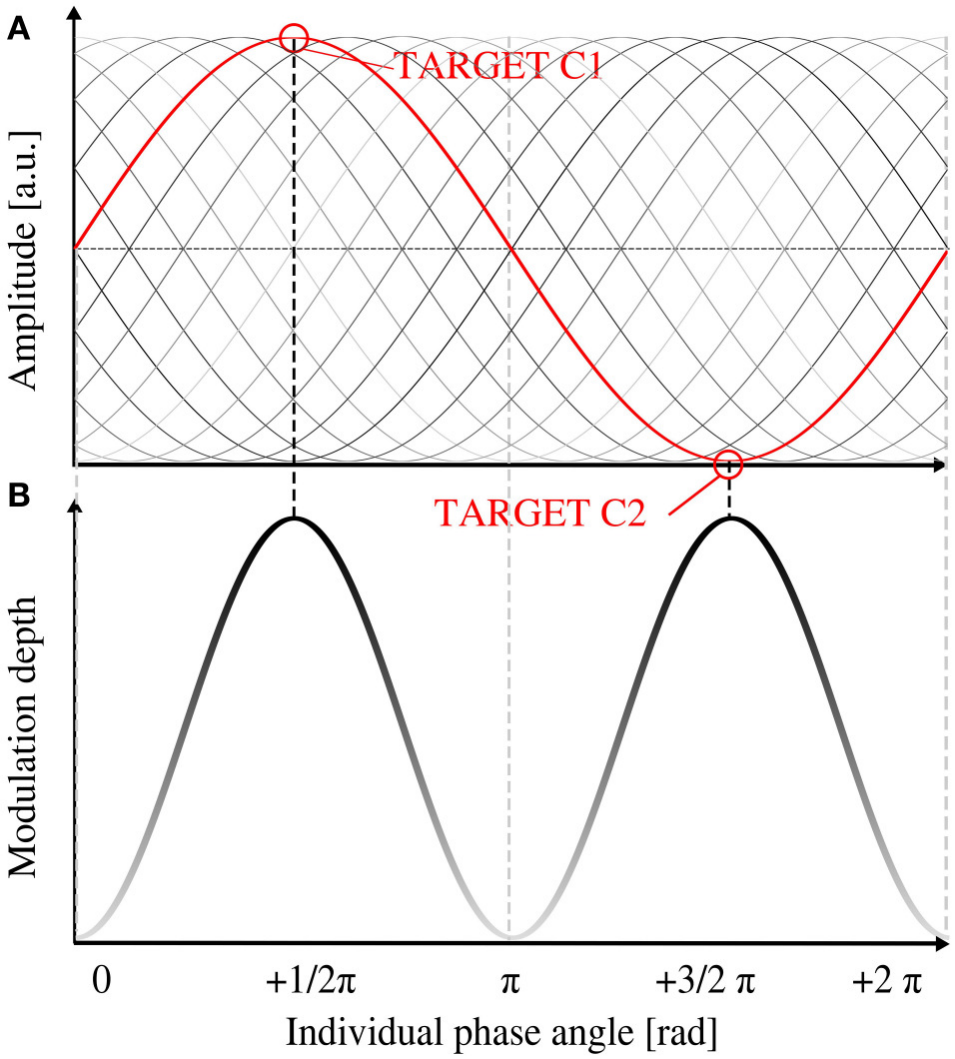
**Relationship between individual phase shift and expected behavioral modulation depth. (A)** Schematic image of the individual alpha oscillation (in gray shades) in relation to the stimulation phase (depicted in red). Black sine represents the expected optimal shift for an increased modulation depth **(B)**. With increasing brightness of the shading, modulation depth decreases. At a phase angle of 1/2 π and 3/2 π at target onset (equals a shift of 0 or π from stimulation phase), target presentation 1 (90°) would coincide with the peak (or trough, respectively) of the EEG peak of the alpha phase (red graph in **A**) and lead to a maximum behavioral modulation depth. No hypothesis was drawn for the polarity (peak or trough) of the EEG oscillation, thus two peaks of modulation depth per alpha cycle were predicted.

Thus, we expected the behavioral modulation depth to depend on the EEG phase at target onset. The modulation depth should increase when targets coincided with the maximum and the minimum (R1 and R2) of the EEG oscillatory wave and decrease if targets were presented more distant from the extrema. Hitting the minimum and maximum of the EEG wave could happen at two instances: R1 target presentation at the peak and R2 target presentation at the trough (see Figure 1, right panel) or vice versa. As a consequence, two possible phases at each of the two target onsets were expected to lead to a high modulation depth (1/2 π and 3/2 π at R1). The two instances where both targets R1 and R2 coincide with a zero crossing (0 and π at R1) were expected to cause a minimal modulation depth (see Figure 4B for visualization). Therefore, we expected a sinusoidal relationship between the ITC and the behavioral modulation depth. The sinusoidal model thus peaked twice per period. R1 was presented at 90° (1/2 π, by definition), thus the −1/2 π term in the model corrects for this offset.

$$\begin{array}{l} y=b_{0}\;+\;b_{1}*\;\sin\;\left(2\;*\;\Phi \;-\;\frac{1}{2}\pi \right)\\ b_{0}=offset,\\ b_{1}=amplitude\;of\;modulation\;depth,\\ \Phi =\frac{t}{2\pi },with\;t=time;individual\;phase\;angles\;of\;all\;subjetcs\\ at\;target\;onset\;\left(R1\right). \end{array}$$

#### Comparison of data from Experiment 1 and 2

The modulation depth was calculated subject-wise for participants in the second experiment as described above. In order to show that the modulation depth was increased during increased stimulation intensity, we compared the two means of Experiments 1 and 2 using a one-sided *t*-test for unequal variances (*ttest2* in MATLAB®). Due to the unequal sample sizes this test applies Satterthwaite's approximation for the effective degrees of freedom (Kreyszig, 2016).

The effect sizes were calculated via the formula of Cohen's *d* (Cohen, 1988).

## Results

We here aimed to investigate whether the visual rhythmic flicker stimulation modulates the perception threshold of brief visual targets via entrainment and, if so, whether this modulation differs fundamentally from perception during arrhythmic stimulation.

### Experiment 1

The modulation depth was the behavioral outcome combining the four conditions (of the factors flicker regularity and target position) and reflects the modulation of phase-dependent perception due to differences in flicker regularity (see Methods Section and supporting information (Figure S1) for derivation of the formula for the modulation depth).

**Figure 5:**
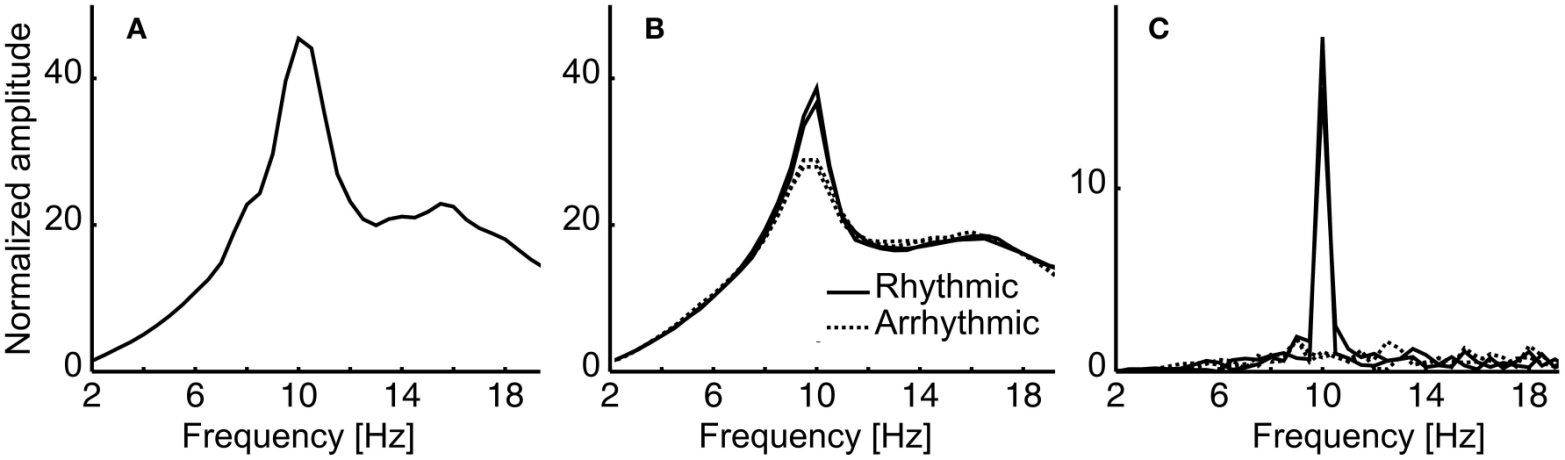
**Amplitude spectra of the resting state and stimulation data, averaged over subjects**. **(A)** Resting state eyes closed **(B)** Induced spectrum for the two rhythmic stimulation conditions (**R1, R2**; solid lines) and the arrhythmic conditions (**A1, A2**; dashed lines). Note that amplitudes (multiplied by frequencies to correct for 1/f bias) cannot be compared to resting state (eyes closed) as stimulation data were collected with eyes open. **(C)** Evoked spectra for the four conditions. Please refer to the Methods Section for further details.

In the first step, amplitude spectra of the stimulated periods and the resting state data were calculated and averaged over subjects. The averaged resting state spectra reveal slightly enhanced amplitudes (normalized amplitudes in the figure reveal 1/f corrected amplitudes) compared to the induced stimulated sequence (Figures 5A,B). This is because resting state was measured with eyes closed, which generally results in higher amplitudes as compared to eyes open. As expected, rhythmic stimulation resulted in a peak at 10 Hz, whereas arrhythmic stimulation shows a rather unspecific elevated spectrum in the area of 10 Hz (Figure 5B, induced spectrum). In the evoked spectrum (Figure 5C), where EEG sequences were first averaged, the 10 Hz peak cancels out in the arrhythmic conditions (A1/A2) but shows a clear peak in the rhythmic conditions (R1/R2).

In the second step we validated this measure. We predicted that with stronger entrainment during rhythmic stimulation, modulation depth should increase. The ratio of the 50% detection threshold during A1 (arrhythmic stimulation, target position at 90°) and the 50% detection threshold during A2 (arrhythmic stimulation, target position at 270°) varied strongly between subjects (between 0.75 and 1.63) and served as a control on a single-subject level. The stronger the deviation of the ratio R1/R2 from A1/A2 (target position 90°/270°) on a single subject level, the greater the modulation depth.

**Figure 6:**
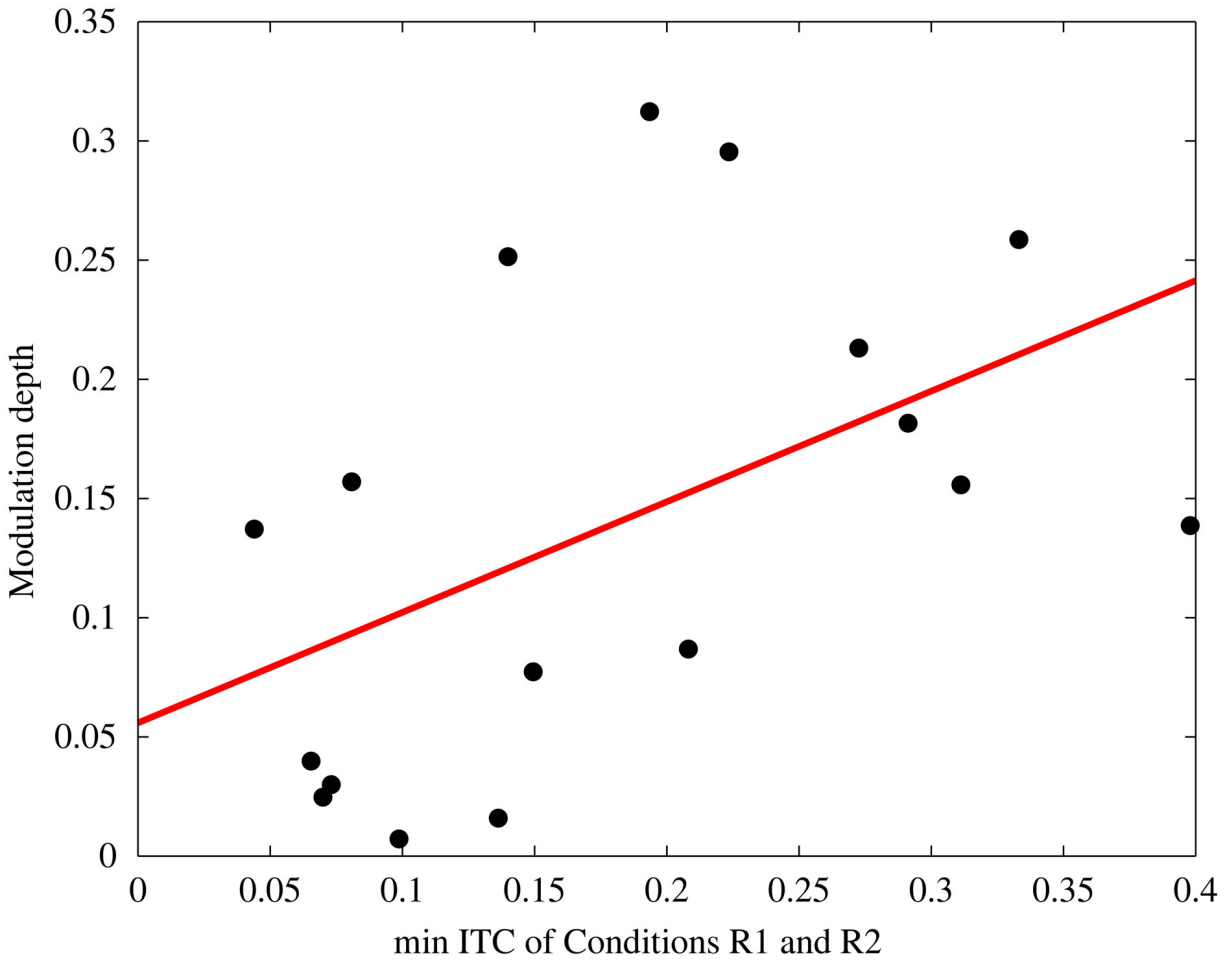
**Correlation between Inter-trial coherence (minimum ITC of the rhythmic conditions) and the behavioral modulation depth (*R*^2^ = 0.25, *p* = 0.04)**. Each data point depicts one subject.

If indeed entrainment was the causal reason for alternations of the detection threshold, then an electrophysiological measure of entrainment, such as the inter-trial coherence (ITC) during rhythmic stimulation should correlate with the modulation depth. Figure 6 depicts the relationship between these two factors, ITC and behavioral modulation depth (*R*^2^ = 0.25, *p* = 0.04). Subjects with low ITC, thus less entrainment, show a modulation depth close to zero. Subjects with high ITC, on the other hand, reveal a stronger modulation depth, thus show differences in the detection threshold during rhythmic compared to arrhythmic stimulation. This correlation could not be found for ITC during arrhythmic stimulation (*R*^2^ = 0.03, *p* = 0.49). The relationship of ITC during rhythmic stimulation and the modulation depth is, however, although significant, comparably small (*R*^2^ = 0.25) and further variance can be explained by the subjects individual EEG phase shift (Henry and Obleser, 2012). Therefore, the subjects' mean phase angle at target onset (R1) was determined.

**Figure 7:**
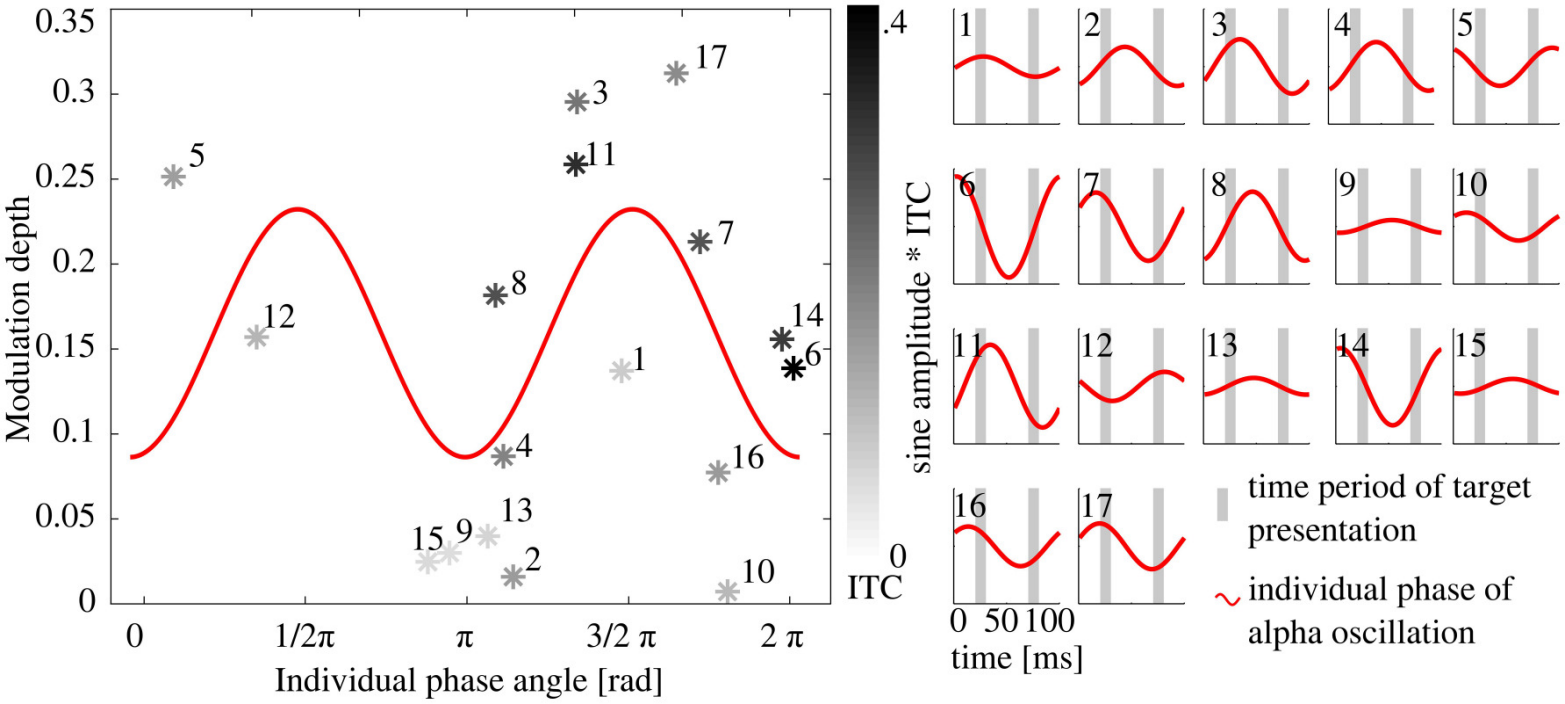
**Relationship between the individual alpha phase angle at target onset and the Modulation depth**. **Left:** Each data point represents one subject's Modulation depth as a function of the individual phase angle at target onset in R1; gray shading of the data points depicts the ITC with black for the maximal ITC and light gray for minimal values. The red 2 Hz sine represents the best fit with estimated parameters offset (b_0_) and amplitude (b_1_), see Methods Section. **(A)** Phase angle of 1/2 π and 3/2 π caused a maximum behavioral modulation depth as both extremes of the EEG oscillation coincide with a target presentation. Numbers refer to the panels in **(B)**, where a sine is plotted schematically with the individual alpha phase angle at target onsets for each subject. The amplitude of the 17 sines (**right** panels) represents the individual ITC, the gray bars mark the time periods of target presentations in R1 and R2. Subjects where both targets were presented at the extremes of the sine and with high ITC have a higher modulation depth (e.g., subject 11), whereas for subjects with a phase angle of 0, π or 2 π target presentation coincided with the zero-crossing of the sine in both conditions (R1 and R2, e.g., subject 6), independently of the ITC, which results in a rather low modulation depth.

We then hypothesized, that an individual alpha phase angle at target onset of ½ π or 3/2 π was optimal for the modulation depth (Figure 4), because these two phases reflect the peak (or trough, respectively) of a sine and lead to maximal modulation depth (see Figure 1). An alpha phase angle of 0 as well as π at target onset, on the other hand, is considered as least optimal, because in that instance both targets would be presented during zero crossing of the EEG oscillation (Figure 4A). Based on this assumption, we fitted a sine with two peaks per period to the modulation depth of the 17 subjects as a function of the individual phase at target onset (R1) with a fixed phase shift of ½ π (as described above). The model was found to significantly describe the relationship between phase at target onset [Figure 7A, *F*_(1, 15)_ = 5.28, *p* = 0.036, *R*^2^ = 0.26, compare also prediction in Figure 4B]. The following estimates resulted from the fitted function: b_0_ = 0.16 ± 0.02 and b_1_ = 0.07 ± 0.03, resulting in the following model function

$$\begin{array}{l} y\;=\;0.16\;+\;0.07\;*\sin\;\left(2\;*\;\Phi \;-\frac{1}{2}\pi \right) \end{array}$$

In Figure 7B, 17 panels additionally show the alpha phase during both target presentations R1 and R2 as well as the amplitude as a relative measure of the ITC. On a subject level, it is shown that ITC as well as individual phase angle at target onset influence the modulation depth.

### Experiment 2

In the second experiment, nothing but the visual flicker intensity in all four conditions was altered. Instead of a flicker at 118 cd/m^2^, we presented the flickering stimulus with 262 cd/m^2^. Most probably due to an altered pupil size (Troland, 1917; Spring and Stiles, 1948; Watson and Yellott, 2012), we found increased detection thresholds for all four conditions (see Table 1 for details).

**Table 1:** **Mean 50% thresholds averaged over subjects [17 for Experiment 1 (Exp. 1) and 14 for Experiment 2 (Exp. 2)]**.

	**Flicker regularity**				
		**Rhythmic**		**Arrhythmic**	
		**Exp. 1**	**Exp. 2**	**Exp. 1**	**Exp. 2**
Target position	Peak (90°)	0.49 (0.04)	0.64 (0.17)	0.56 (0.04)	0.73 (0.19)
	Trough (270°)	0.44 (0.03)	0.53 (0.14)	0.50 (0.03)	0.63 (0.17)

Standard error of the mean (SEM) given in parenthesis.

**Figure 8:**
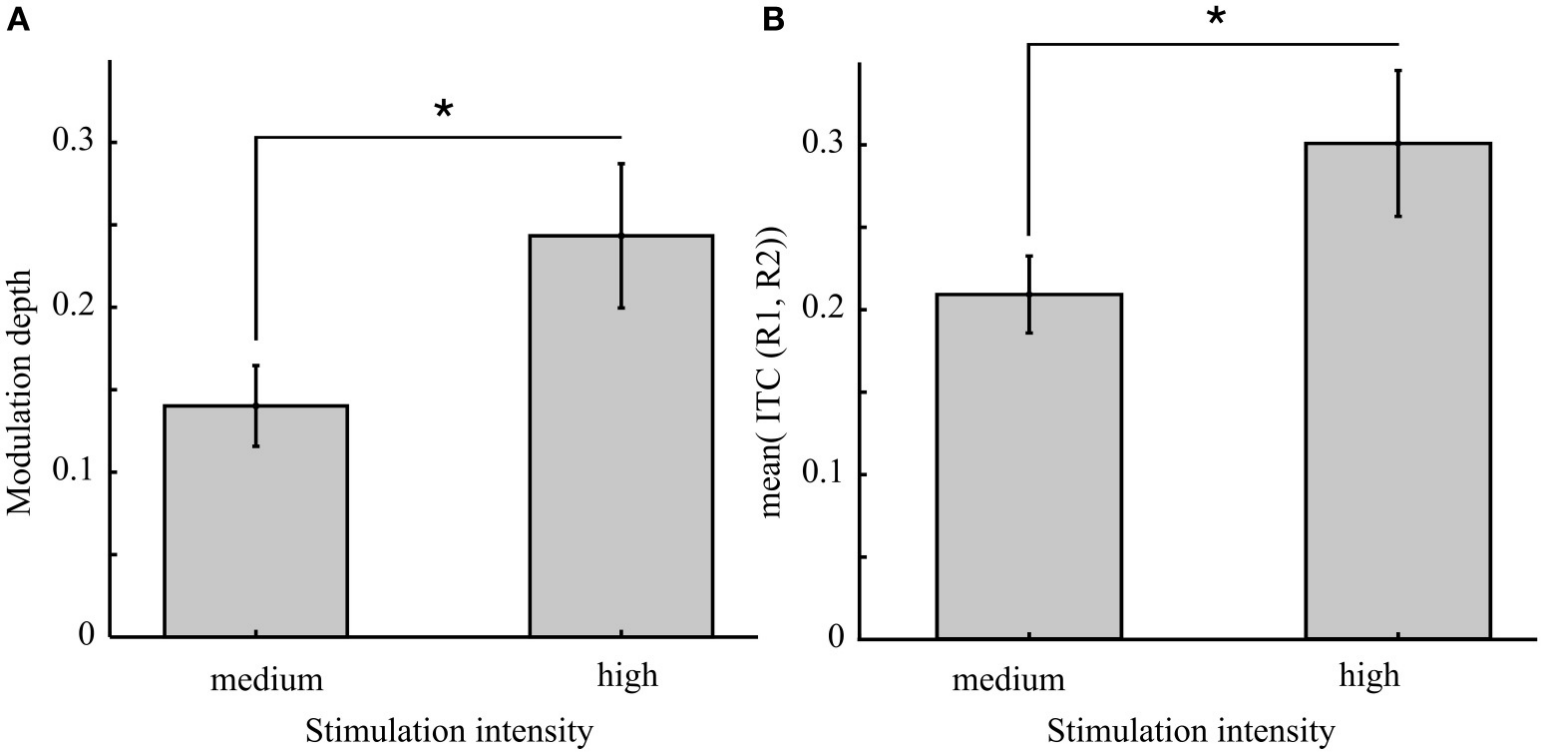
**Increased light intensity causes a greater behavioral modulation depth which is suggested to result from stronger inter-trial coherence (ITC)**. **(A)** The mean modulation depth from conditions 1 and 2 (rhythmic stimulation) compared between the two experiments [medium (Exp. 1) and high (Exp. 2) light intensity of the flicker] revealed a significant increase [*t*_(19.5)_ = 1.9, *p* = 0.037] as well as the ITC [*t*_(20)_ = 1.83, *p* = 0.041] shown in **(B)**. Asterisks indicate the 0.05 significance level. Error bars reflect the *SEM*.

Based on the concept of the Arnold tongue, an increased light intensity is expected to lead to stronger phase locking. Indeed we found an increased inter-trial coherence using a one-sided *t*-test for unequal variance [Figure 8, right panel; *t*_(20)_ = 1.83, *p* = 0.041, *d* = 0.68]. As a consequence, the modulation depth was expected to increase. Our results show that modulation depth is increased during stimulation at increased light intensity [mean modulation depth and standard error at medium intensity: 0.14 ± 0.02 vs. high intensity: 0.24 ± 0.05; *t*_(19.5)_ = 1.9, *p* = 0.037, *d* = 0.71].

### Resting state

In order to investigate whether the resting state oscillation frequency relates to the strength of entrainment, we determined the peak frequency in the alpha range for every subject prior to the experiment. The overall mean frequency was 10.09 ± 1.46 Hz. The mean individual distance from 10 Hz was 0.91 ± 1.12 Hz. After median split of the minimum ITC of conditions R1 and R2 per subject, for the resulting two groups the mean individual alpha frequency distances from 10 Hz were determined. No significant differences were found using a signed rank test [*z* = 0, *p* = 1].

## Discussion

In the present study, we found evidence for rhythmic visual stimulation to elicit entrainment. In line with the concept of the Arnold tongue, which predicts the response of an oscillator during entrainment, we found that rhythmic and arrhythmic stimulation led to differing modulations of perception, suggesting different underlying mechanisms for the two types of stimulation.

### Behavioral measure of the modulation depth

The occipito-parietal alpha phase has been shown to modulate visual detection (Klimesch et al., 2007). Based on these findings, we here designed a behavioral paradigm to investigate the underlying mechanism of SSVEPs. We therefore introduced a new measure of modulation depth that includes arrhythmic and rhythmic stimulation at two opposing phase angles. Due to a number of confounds that necessarily appear in visual detection tasks during visual stimulation (target contrast, temporal attention shifts, pupil size; see below), the absolute detection thresholds of the four conditions (factors flicker regularity and target position) are not suitable for a direct comparison (see also Data sheet S1).

Our results show that the modulation depth increased with increasing inter-trial coherence (ITC) as a measure of entrainment in the rhythmic condition. The ITC in the arrhythmic condition appears not to be linked to the modulation depth. In other words, subjects that showed stronger entrainment also had a higher modulation depth of their behavioral performance, whereas subjects that could not be entrained by the external flicker (low ITC) revealed a behavioral modulation depth close to zero (Figure 4). A modulation depth close to zero reveals either the fact that arrhythmic and rhythmic stimulation did not induce different effects on relative target detection or that the individual EEG phase angles did not differ at the two target onsets of R1 and R2 (see below). This result is in line with previous studies that reported phase dependent behavioral performance as a consequence of visual entrainment (Mathewson et al., 2011; de Graaf et al., 2013; Spaak et al., 2014), which evaluates our measure of behavioral modulation depth.

Additionally, we determined the alpha phase angle at 10 Hz during target onset. Brain responses of the parietal cortex show an individual shift between EEG and stimulation phase as it has previously been described for the auditory system (Henry and Obleser, 2012). We found this individual shift in our data to predict the modulation depth: if the two target presentations (90° and 270°) coincided with local maxima and minima of the alpha oscillation, perception was modified maximally (high modulation depth). Target presentations near or at the zero crossing of the alpha oscillation (0, π, 2π) led to rather similar detection thresholds at the two target positions (see schematic explanation in Figures 1, 5 and the sinusoidal fit in Figure 7).

Furthermore, for subjects with low entrainment (small ITC) the determined phase angle is rather coincidental and not a valid predictor for the modulation depth. In conclusion, both factors, inter-trial coherence and phase angle at target onset play a crucial role in the behavioral modulation depth. Altogether, the ITC and the alpha phase angle at target onset validate the, to our knowledge, newly introduced measure of the modulation depth.

Likewise, the different contrast levels at which targets were presented in conditions R1 and R2 did not bias our results, as the relative measure (R1/R2) was corrected by subtracting the equivalent ratio of the arrhythmic stimulation (A1/A2). Thus, this confound was eliminated from our behavioral measure as well.

### Superposition or entrainment?

Based on these findings we further conclude that phase dependent detection modulation during rhythmic stimulation is not based on phase reset produced by an ERP (Hanslmayr et al., 2007b; Capilla et al., 2011). During arrhythmic stimulation the flicker stimuli around the target presentation were kept constant and identical to the rhythmic condition (gray boxes in Figure 2). A potential ERP-elicited phase-reset would thus be the same for both flicker regularities. As a conclusion, an ERP-elicited alpha synchronization cannot bias our results, because if occurring at all it would be present in both types of stimulation and thus result in a modulation depth at around zero.

As a conclusion, it seems likely that SSVEPs reflect an entrained alpha rhythm. Neural populations oscillate between states of high and low excitability in resting state and have been shown to predict the likelihood of a visual stimulus to be perceived (Hanslmayr et al., 2007a). These neural populations show more or less phase and frequency synchronized activity, with higher synchronization during decreased attention and less synchrony when attention is focused on perception (Cooper et al., 2003). The flicker is suggested to entrain this ongoing oscillator or enhance the amplitude by forcing neural populations to oscillate at the external frequency. This induces a predictable detection of targets, presented at specific phases of this entrained alpha rhythm.

### Entrainment—a mechanism purely based on temporal attention?

However, Mathewson et al. (2012) found that the effect of phase dependent detection can also be gained via arrhythmic stimulation. As our measure only describes a difference of the relative detection modulation during arrhythmic and rhythmic stimulation, we cannot exclude that detection is modulated during arrhythmic stimulation as well, but to a lower extent, which is why Experiment 2 was performed.

Temporal attention describes a mechanism that prepares the brain for upcoming events after an expected time window (Barnes and Jones, 2000; Nobre et al., 2007) and entrainment is often described as a mechanism that creates temporal attention (Lakatos et al., 2008; de Graaf et al., 2013). Likewise, Mathewson et al. (2012) described temporal attention as the underlying mechanism of entrainment. In their study, targets were presented after a rhythmic or an arrhythmic flicker sequence. The (average) frequency of the flicker sequence enhanced perception of targets presented at an inter-trial interval that equals that of the previous flicker (or the average of the inter-trial interval, respectively). In order to find out if indeed arrhythmic stimulation may cause entrainment but to a weaker extent, we here intended to investigate whether, beyond temporal attention, the two types of stimulation elicit different fundamental mechanisms in the brain. Compared to Mathewson et al. (2012), who presented targets subsequent to a fixed stimulation period of 576 ms with a jittered interval of 36–177 ms, in our study temporal attention was strongly reduced due to the continuous block paradigm. Targets were presented every 3–5 s during a 5 min flicker block.

On the millisecond scale, temporal attention may arise from flicker regularity (Barnes et al., 2005; Lakatos et al., 2008; Stefanics et al., 2010). This confounding factor was eliminated by the ratios of the 50% thresholds within the factor flicker regularity (R1 divided by R2 and A1 divided by A2).

The phase dependent performance modulation reported by Mathewson et al. (2012), however, reflects amplitude differences of their behavioral measure between rhythmic and arrhythmic stimulation as well and we cannot exclude that temporal attention, even though reduced to a minimum, may have biased our results (Lasley and Cohn, 1981). Interestingly, Schnuerch et al. (2013) showed, that temporal attention and stimulus intensity do not interact, thus stronger stimulation intensity is not expected to result in stronger temporal attention.

### Increased stimulation intensity causes increased modulation depth along with stronger entrainment

Thus, we performed a second experiment with the exact same parameters but with increased light intensity for the flicker in both the rhythmic and the arrhythmic condition. Based on the findings reported by Schnuerch et al., 2013), a difference in behavioral performance cannot be explained by temporal attention. Furthermore, if both rhythmic and arrhythmic stimulation revealed a superposition of ERPs, changes in the perceptual threshold would change equally for all four conditions and result in a similar behavioral modulation depth, independently of the flicker intensity.

In line with the concept of the Arnold tongue (Pikovsky et al., 2003) we here found a significant increase of the modulation depth when the stimulation intensity was increased. As a function of stimulation frequency and intensity, the Arnold tongue predicts the brain responses of the parietal cortex during external stimulation in case entrainment is the underlying mechanism (Notbohm et al., 2016).

Thus, our finding of increased entrainment (determined via ITC) and an increased behavioral modulation depth in Experiment 2 is interpreted as evidence for both stimulation types causing different mechanisms in the brain. As possible explanation, we suggest that during rhythmic stimulation the intrinsic alpha oscillator is entrained to the external stimulation in a way that cannot be explained purely by temporal attention but may include a bottom-up component that entrains alpha oscillations in addition to attentional modulation (Thut et al., 2006), that has also been shown to interact with the SSVEP (Müller et al., 2003). While arrhythmic stimulation may induce temporal attention effects, we suggest that this bottom-up component of entrainment is not induced via arrhythmic stimulation.

### The role of the ongoing alpha oscillations

Here, we stimulated all subjects at 10 Hz, independently of their individual alpha frequency. This was mainly due to technical reasons: adjusting the paradigm to the individual alpha frequency would mean that either the flicker on- and off-times would have to differ between subjects (at a refresh rate of 100 Hz, 9 Hz (ca. 111 ms per cycle) can only be produced by e.g., 50 ms (five screens) flicker on and 60 ms (six screens) flicker off (= 9.09 Hz) or the refresh rate would have to alter between subjects, which would then result in different durations of target presentation. We decided to avoid this confound at a cost of differing distances to the individual alpha frequency, which was found to be rather low. Assuming these limitations were not existent, stronger entrainment would be expected altogether, especially for those subjects with greater deviance of the IAF from 10 Hz.

### Disentangling temporal attention and entrainment

Based on the comparison of the modulation depth between Experiment 1 and 2, we found that rhythmic and arrhythmic stimulations produce fundamentally different responses in the brain. While rhythmic stimulation produces an SSVEP, which is suggested to be based on entrainment, arrhythmic stimulation was not found to substantially interact with the ongoing alpha oscillation. The newly introduced behavioral measure (the modulation depth) allowed us to compare perception at different stimulation intensities. The different stimulus intensities used in Experiment 2 resulted in different degrees of modulation depth which cannot be explained by temporal attention.

## Future directions

The individual differences of entrainment (ITC variations) suggest future investigations with respect to top-down control of alpha-oscillations (Haegens et al., 2011a; Wang et al., 2016). Does an increased connectivity of the visual cortex with areas like the frontal cortex that exhibit top-down influence (Buschman and Miller, 2007) impair or improve entrainment? This would provide further insight into directionalities of entrainment (Sokoliuk and VanRullen, 2016).

Future studies may also apply the newly introduced measure of the behavioral modulation depth to test stimulation at different distances from the intrinsic alpha frequency. While comparisons of the absolute thresholds would again be confounded by different backward and forward masking effects due to altered distances between target and flicker (due to different inter-trial intervals), the introduced measure remains unaffected of this confound. Based on the predictions made by the concept of the Arnold tongue, at ~8 or 12 Hz for instance, entrainment should be less likely to appear (for a subject with an individual alpha frequency at 10 Hz). As a consequence, a modulation depth closer to zero would be expected. The same prediction would hold for an even lower light intensity for the flicker stimulus as used in Experiment 1.

## Conclusion

We here introduced a new measure of behavioral modulation depth that allowed us to investigate the impact of rhythmic visual stimulation on perception modulation. Alpha oscillations were shown to modulate perception in a phase-dependent manner (Klimesch et al., 2007). Here, we found that rhythmic compared to arrhythmic stimulation led to significantly different modulations of this phase-dependent perception. In line with the concept of entrainment, we conclude that a rhythmic but not an arrhythmic visual flicker can entrain the alpha oscillations in the parietal cortex, while temporal attention can be produced by both types of stimulation. In the present study, we showed that entrainment is possible at or very close to the individual alpha frequency (10 Hz stimulation). The introduced study paradigm can be applied in future studies to investigate whether we can shift alpha-dependent perception toward different surrounding frequencies, as predicted by psychophysical studies (Pikovsky et al., 2003; Notbohm et al., 2016). A comprehensive evaluation of alpha entrainment via sensory stimulation will provide further implication also for clinical relevance.

## Author contributions

CH and AN designed research; AN performed research; AN analyzed data; CH and AN wrote the paper.

## Funding

This work was supported by grants of the Ph.D. programme “Signals and Cognition” and the Excellence Cluster “Hearing4All” (EXC1077).

### Conflict of interest statement

The authors declare that the research was conducted in the absence of any commercial or financial relationships that could be construed as a potential conflict of interest.

## Supplementary material

Figure S1 **Individual behavioral performance. (A)** Single subject performance is shown in black, average performance in red. **(A)** Left panel: 50% detection threshold at target position 90° (R1) and 270° (R2) during rhythmic stimulation (R). Central panel: detection thresholds during arrhythmic stimulation at target position 90° (A1) and 270° (A2). Right panel: Relative performance [target position 90° (1) divided by target position 270° (2)] for rhythmic (left) and arrhythmic (right) stimulation. Due to the large variability between subjects, average values are not representative. The modulation of perception during rhythmic compared to arrhythmic stimulation must be determined on a single subject level. Error bars show standard deviations. **(B)** Two confounding factors, temporal attention and difference in visual contrast level need to be considered and are eliminated in our behavioral measure of the modulation depth.
