# Supplementary figures and images for: Flicker Regularity Is Crucial for Entrainment of Alpha Oscillations
Source: Front Hum Neurosci. 2016 Oct 13;10:503. doi: 10.3389/fnhum.2016.00503 (PMC5061822; doi:10.3389/fnhum.2016.00503)

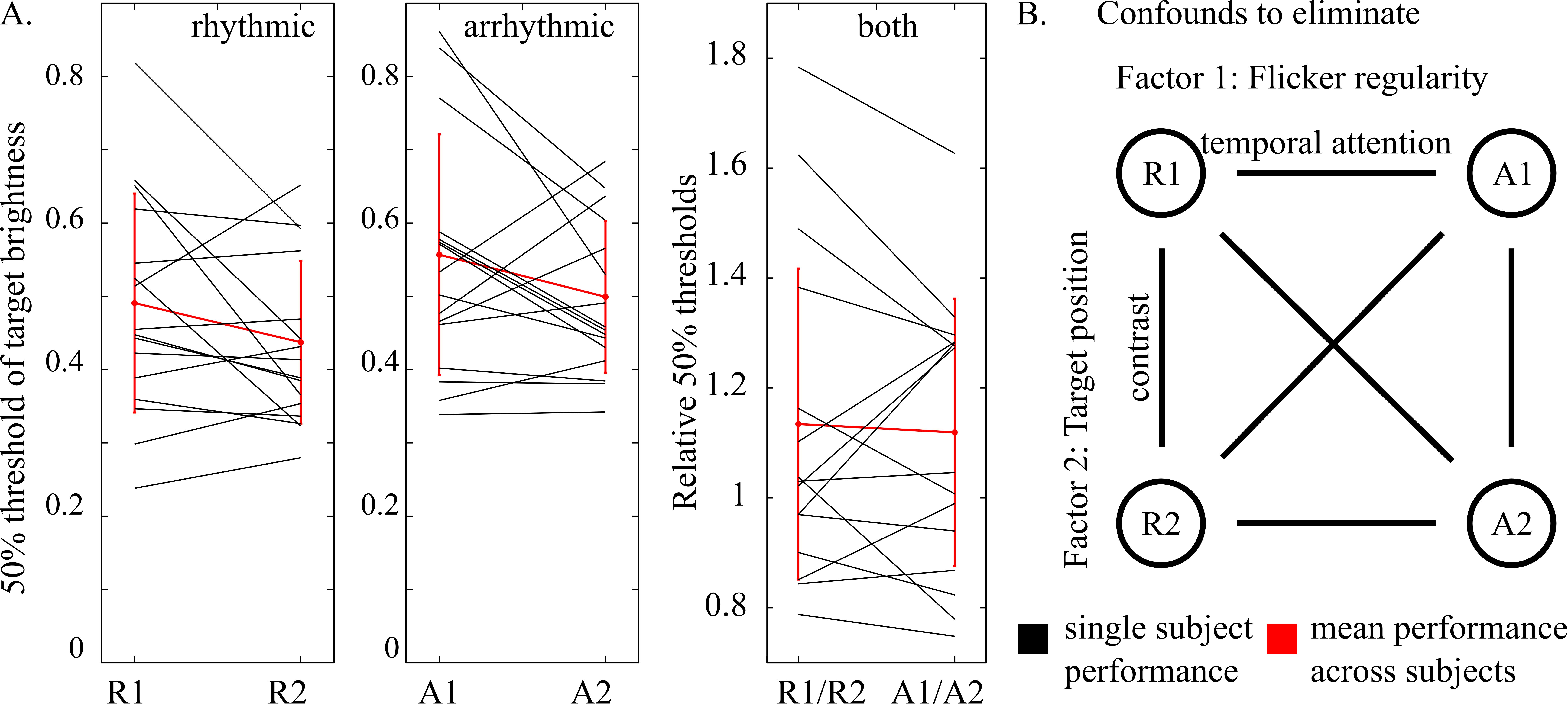

Supplement: Figure S1 — Individual behavioral performance. (A) Single subject performance is shown in black, average performance in red. (A) Left panel: 50% detection threshold at target position 90° (R1) and 270° (R2) during rhythmic stimulation (R). Central panel: detection thresholds during arrhythmic stimulation at target position 90° (A1) and 270° (A2). Right panel: Relative performance [target position 90° (1) divided by target position 270° (2)] for rhythmic (left) and arrhythmic (right) stimulation. Due to the large variability between subjects, average values are not representative. The modulation of perception during rhythmic compared to arrhythmic stimulation must be determined on a single subject level. Error bars show standard deviations. (B) Two confounding factors, temporal attention and difference in visual contrast level need to be considered and are eliminated in our behavioral measure of the modulation depth. [file Image1.JPEG]
