# Supplementary material for: Flicker Regularity Is Crucial for Entrainment of Alpha Oscillations
Source: Front Hum Neurosci. 2016 Oct 13;10:503. doi: 10.3389/fnhum.2016.00503 (PMC5061822; doi:10.3389/fnhum.2016.00503)
Supplement: Supplementary file 2 [file DataSheet1.DOCX]

S1. Derivation of the introduced measure of the behavioral modulation depth

The 50% detection threshold was on average lower during rhythmic flicker (Condition R1 and R2; Figure S1A, left panel) and when targets were presented in the gap between two flicker-stimuli (R2/A2, target position 270°, Figure 1SA, two leftmost panels) as compared to presentation together with the flicker stimulus (R1/A1, 90°).

However, a 2x2 ANOVA neither revealed significant results for the main effects (Flicker regularity: F(1,15)= 3.75, p = 0.06 and Target position: F(1,15) = 2.94, P = 0.09) nor for the interaction (F(1,30) = 0.01, p = 0.94). The expected missing effects result from a large inter- subject variability as shown in Figure S1A. Six out of the 17 subjects had lower detection thresholds during A1 (arrhythmic stimulation, target with flicker) as compared to A2 (target presentation in the gap between two flicker stimuli).

If targets are presented together with the surrounding entraining stimulus (see Figure 2 for stimulus introduction), the contrast between stimulus and surrounding is much lower as in condition 2, where the target stimulus, presented in the gap between two stimuli, is presented on a surrounding black screen). A lower threshold means improved performance and one would expect detection rates to be higher (low 50% threshold) if contrast is high. This has been the case for 11 out of 17 subjects, but as mentioned earlier, for six the inverse was found.

Therefore, we took the individual relative detection threshold (A1 divided by A2) as a control term. This way, the individual relative detection threshold during arrhythmic stimulation is determined and serves as a control for the relative detection threshold during rhythmic stimulation (R1 divided by R2).

On average, again the two relative values R1/R2 (rhythmic stimulation at target positions 90° and 270°) and A1/A2 (arrhythmic stimulation at target positions 90° and 270°) did not differ (1.13± 0.28 for R1/R2 and 1.12±0.24 for A1/A2, see Figure S1A, right panel). This is due to the fact that the direction of the effect differs largely between subjects (7 out of 17 subjects show a larger relative threshold during arrhythmic stimulation, 10 subjects show the inverse effect). A comparison over subjects of the two relative values is not representative, because entrainment can affect perception modulation in both directions (see explanation on individual phase delay and Figure 5&6 for further information).

We hypothesized, that in case rhythmic stimulation does not influence the perception threshold (compared to arrhythmic) then the relative thresholds (Figure S1A, right panel) should remain unaffected. Therefore, the absolute difference of the two relative thresholds on a subject level was considered as modulation of baseline detection. Thus, a modulation depth different from zero reflects an alteration of perception due to flicker regularity.

Moreover, this calculation eliminates the two confounding factors of target contrast differences and temporal attention, which is expected to differ between rhythmic and arrhythmic conditions (Figure S1B).
